# Cryo-electron microscopy of an extremely halophilic microbe: technical aspects

**DOI:** 10.1007/s00792-016-0912-0

**Published:** 2017-01-03

**Authors:** Daniel Bollschweiler, Miroslava Schaffer, C. Martin Lawrence, Harald Engelhardt

**Affiliations:** 1 Max-Planck-Institut für Biochemie, Am Klopferspitz 18, 82152 Martinsried, Germany; 2 Department of Biochemistry, University of Cambridge, Cambridge, UK; 3 Department of Chemistry and Biochemistry, Montana State University, Bozeman, MT USA

**Keywords:** *Halobacterium salinarum*, Haloarchaea, Gas vesicles, Cryo-electron tomography, Focused-ion-beam micromachining, Vitrification

## Abstract

Most halophilic *Archaea* of the class *Halobacteriaceae* depend on the presence of several molar sodium chloride for growth and cell integrity. This poses problems for structural studies, particularly for electron microscopy, where the high salt concentration results in diminished contrast. Since cryo-electron microscopy of intact cells provides new insights into the cellular and molecular organization under close-to-live conditions, we evaluated strategies and conditions to make halophilic microbes available for investigations in situ. *Halobacterium salinarum*, the test organism for this study, usually grows at 4.3 M NaCl. Adaptation to lower concentrations and subsequent NaCl reduction via dialysis led to still vital cells at 3 M salt. A comprehensive evaluation of vitrification parameters, thinning of frozen cells by focused-ion-beam micromachining, and cryo-electron microscopy revealed that structural studies under high salt conditions are possible in situ.

**Electronic supplementary material:**

The online version of this article (doi:10.1007/s00792-016-0912-0) contains supplementary material, which is available to authorized users.

## Introduction

Cryo-electron microscopy and cryo-electron tomography are successful techniques to investigate the structure and molecular organization of eukaryotic cells, intact bacteria, and archaea at close-to-live conditions (Baumeister 2015; Plitzko 2009). The availability of direct electron detectors and a new type of phase plate for transmission electron microscopes improve the resolution and visibility of structural details considerably (McMullan et al. 2014; Danev et al. 2014; Fukuda et al. 2015). These technical innovations further close the gap between molecular and cellular structure research as recent results of in situ cryo-electron tomography demonstrate (Khoshouei et al. 2016; Mahamid et al. 2016).

But there are still obstacles in the course of sample preparation for cryo-electron microscopy (cryo-EM) that may affect the native macromolecular organization or even the integrity of cells. Characteristic parameters of the organisms´ natural or experimental environment, such as the temperature and composition of the growth medium, should ideally remain unchanged until vitrification. Although not frequently applied, moderate conditions are technically controllable during preparation, i.e. physiological temperatures around 37 °C for microbial and mammalian cells, and common isoosmotic media for various organisms. However, temperatures around 100 °C for hyperthermophilic archaea, or hypersaline media containing sodium chloride close to the saturation limit for extremely halophilic microbes are clearly detrimental or even inapplicable to sample preparation in cryo-EM. While thermophiles can be incubated at moderate temperatures prior to vitrification as a technical compromise, halophiles lyse in low salt conditions. Attempts to study extremely halophilic microorganisms by electron microscopy thus usually include cross-linking and chemical embedding (Robertson et al. 1982; Trachtenberg et al. 2000; Ring and Eichler 2001; Keklar et al. 2009; Pietilä et al. 2013) or air drying (Strunk et al. 2011; Fröls et al. 2012), an approach that was already applied in a very early study of *Halobacterium* (Houwink 1956).

In this study, we investigated approaches to make halophilic microbes available for in situ cryo-EM. *Halobacterium salinarum* is an extremely halophilic archaeon and grows in media containing 4.3 M NaCl (Oesterhelt and Krippahl 1983) which is far from common isoosmotic solutions supplemented with ≈0.14 M NaCl for (mostly mammalian) cells. *Halobacterium salinarum* thus constitutes a fastidious test organism. The strategy was to minimize the salt concentration without disturbing the vitality of the cells first and to find suitable conditions for vitrification and microscopy based on the required salinity of cell suspensions. We show that cryo-EM of halobacteria with salt concentrations up to 3 M NaCl is feasible.

## Materials and methods

### Strains, media and growth

*Halobacterium salinarum* strain S9 (Wagner et al. 1981) was originally obtained from the culture collection of the Department of Membrane Biochemistry (D. Oesterhelt), Max Planck Institute of Biochemistry, Martinsried, Germany. The growth medium consisted of 4.3 M NaCl, 81 mM MgSO_4_, 27 mM KCl, 10 mM Na-citrate, and 1% w/v Oxoid peptone (pH 7.1). The inoculum was 10 ml of freshly grown precultures (48 h, OD_600_ ≈0.8) per 1 l of medium for experimental purposes. Cells grew at 37 °C while shaking at 100 rpm in Erlenmeyer flasks and usually were collected in the late exponential growth phase after 72 h at an OD_600_ of ≈0.9.

Swarm agar plates consisted of normal medium and 0.3% w/v agar. The inoculum (10 µl of late growth phase cultures) was placed in the center of the plates and incubated for 5 days at 37 °C. Cells were picked from the edge of the most motile culture out of 5 parallel experiments, inoculated into 50 ml of liquid medium and incubated as usual, inspected for motility in the microscope and used for further selection of motile cells on swarm agar plates. *Halobacterium salinarum* showed a significantly increased swarm motility after 5 cycles (strain S9 SW#5; Online Resource 1) that was indistinguishable concerning motility from samples after 10 cycles.

### Dialysis approach

Aliquots (12 ml) of *H. salinarum* cultures, grown for 60 to 72 h (OD_600_ 0.9 to 1.0), were dialyzed in Slide-A-Lyzer™ bags against a gently stirred solution of 81 mM MgSO_4_ (1 l) at room temperature (RT). Samples of 0.2 ml taken from the cell suspensions during the dialysis experiments were analyzed for the remaining salt and inspected for the integrity of cells by microscopy. The dialysis kinetics had been characterized beforehand. For this a 4.3 M NaCl plus 81 mM MgSO_4_ solution was dialyzed as above and the residual salt analyzed every 7 min. The osmometer OSMOMAT™ 030 (Gonotec, Berlin, Germany) served to determine the osmolality of samples. The measures were calibrated by means of reference solutions containing 81 mM MgSO_4_ and 0 to 4.3 M NaCl to calculate the concentration of NaCl.

*Halobacterium salinarum* intended for growth at lower salt concentrations were collected from cultures containing 4.3 M NaCl, dialyzed as described above against 1 l of medium with the reduced salt content for 2 h and used as inoculum for cultures with corresponding NaCl concentrations. Cell suspensions for cryo-electron microscopy experiments were dialyzed accordingly against a standard solution of 3 M NaCl plus 81 mM MgSO_4_ at RT or 37 °C.

### Vitrification and cryo-electron microscopy

*Halobacterium salinarum* cells (1 ml) were centrifuged at 150 x *g* for 30 min, the supernatant carefully removed and the concentrated cell suspension mixed with 0.1 ml of standard salt solution. 100 µl of colloidal 10 nm-sized BSA-coated gold markers (Aurion) were spun down using a benchtop centrifuge (4000×*g*, 10 min). The supernatant was discarded and the gold marker pellet resuspended in 100 µl 3 M NaCl standard salt solution. 4 µl of this gold marker resuspension and 4 µl of the cell suspension were applied to Quantifoil™ Cu 200 R 2/1 grids that had been exposed to glow discharge for 30 s. After blotting, the samples were plunge-frozen using a home-made plunger (Plitzko and Baumeister 2007) or a Vitrobot™ Mark IV (FEI, Eindhoven, The Netherlands) at RT and inspected in a Tecnai Polara G2 transmission electron microscope (FEI), equipped with a 300 kV field emission gun, a post-column energy filter, and a 2*k* × 2*k* CCD detector (GATAN Inc., Pleasanton, California, USA). Data were recorded under cryo-conditions (about −190 °C) and low-dose exposure (60–120 e^−^/Å^2^ total dose) at primary magnifications of 18,000× or 22,500×.

Focused-ion-beam (FIB) milling for thinning of vitrified samples on grids prior to cryo-EM was performed in a Quanta™ 3D FEG dual beam FIB scanning electron microscope (SEM; FEI, Eindhoven, The Netherlands) under cryo-conditions. Milling was performed by cutting wedges of 15 to 20 µm width into the specimen at an angle of 4°–6°, as described in more detail in (Rigort et al. 2010; Rigort and Plitzko 2015; Schaffer et al. 2016). Accompanying SEM images were recorded at 3 kV acceleration voltage and a beam current of 20 pA at 10,000× magnification for preparation control.

## Results and discussion

High amounts of salt in cell suspensions diminish contrast between the structures of microbial cells and the surrounding medium in cryo-EM. Furthermore, NaCl at concentrations close to the saturation limit is prone to crystallize swiftly, evaporation considerably changes the concentration in small droplets on grids for electron microscopical investigations, and the increased viscosity of the solution has effects on the blotting behavior during sample preparation. These features require carefully adapted vitrification parameters for cryo-EM. To accomplish reasonable starting conditions and to avoid unnecessarily high salt concentrations for electron microscopy, we first attempted to minimize the salinity of media to a point where viability and vitality of *H. salinarum* were not yet impaired.

### Minimal salt conditions for *H. salinarum*

We chose two criteria to identify healthy and viable cells: growth and motility. To increase the relative amount of motile cells, *H. salinarum* strain S9 was grown on swarm agar plates and repeatedly selected for particularly motile populations as described in Materials and Methods. The resulting test strain contained 80–90% actively moving cells at the end of the exponential growth phase (72 h) when inspected in the light microscope. These microbes showed massive flagellation (see Online Resource 1). Shorter (50 h) or longer (90 h) growth was unfavorable since cells were less motile or progressively lysed, respectively.

We cultivated strain SW#5 in media with original and reduced NaCl content to evaluate the lower limit of the cells´ salt tolerance. The Mg^2+^ concentration remained unchanged since *H. salinarum* needs divalent ions for the integrity of its S-layer, which functions as the stable cell wall (Engelhardt 2007, 2016). The strain showed apparently unimpaired growth down to 3 M NaCl but only minimal growth at 2.5 M. This result is in agreement with other observations (Zeng et al. 2006). However, only *H. salinarum* in 3.5 M NaCl medium was indistinguishable from cells in control samples (4.3 M). *Halobacterium salinarum* cultivated with 3.0 M (2.5 M) NaCl showed ≤10% (≥80%) aberrant and ≥70% (>90%) immotile cells. Investigations by Vauclare et al. (2015) revealed that the intracellular KCl content dropped concomitantly with external salt concentrations ≤2.5 M—*H. salinarum* adapts the osmotic pressure by accumulating KCl in the cytoplasm (Oren 2008)—and that the integrity of cytosolic proteins suffered from low salt conditions. According to our results, growth media contained 3.5 M NaCl for subsequent experiments.

**Fig. 1:**
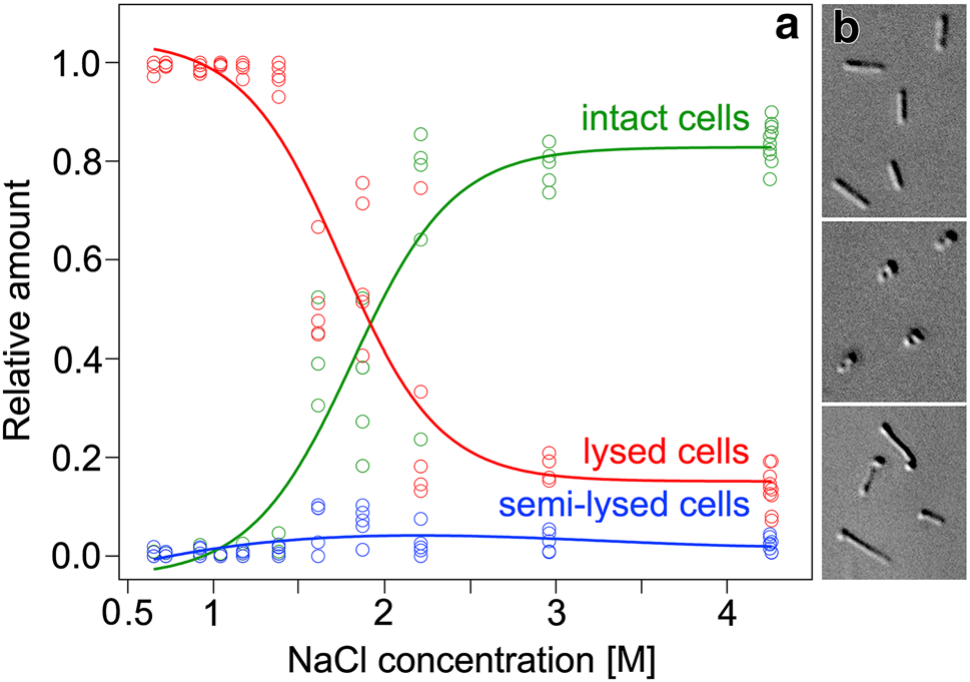
Morphological integrity of *Halobacterium salinarum* in NaCl solutions. Cells were grown in 4.3 M NaCl and 81 mM MgSO_4_ and dialyzed against a solution of 81 mM MgSO_4_. **a** Plot of the relative amount of intact, lysed, and semi-lysed cells with respect to the remaining NaCl concentration after dialysis. Analysis of 60 microscopic frames containing 8139 cells in total. Curve fits were calculated using Igor Pro 6.3 (WaveMetrics®). **b** Selected cells illustrating intact, lysed, and semi-lysed states (from *top* to *bottom*) in the light microscope (DIC imaging)

The approach for further salt reduction was to remove NaCl by dialysis after cell growth. Figure 1 illustrates that the amount of intact cells decreased below 2.5 M but remained almost unchanged to 3.0 M NaCl. The cells were still fully motile and did not show other obvious shortcomings compared to the control without salt reduction. The basic amount of lysed cells (≈10% on average) appears to originate from the long-term culture and may also include false positives. Short-time exposure to lower salinity is indeed less harmful to *H. salinarum* than incubation for many hours or several days (Vauclare et al. 2015). We, therefore, combined cell growth at 3.5 M NaCl (72 h) and short-term dialysis to 3.0 M NaCl at constant 81 mM MgSO_4_ prior to vitrification experiments (total time after dialysis until freezing less than 1 h).

### Plunge freezing of *H. salinarum* in 3 M NaCl

**Fig. 2:**
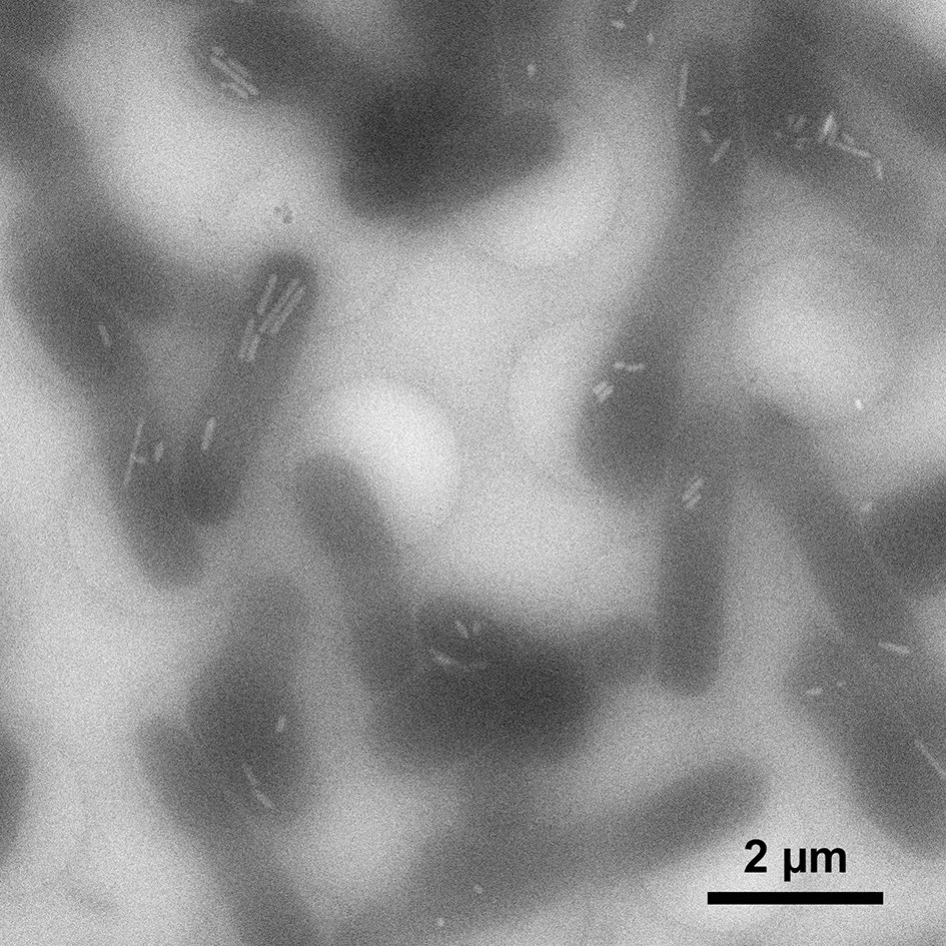
Cryo-electron microscopy of intact *Halobacterium salinarum* cells after vitrification in 3 M NaCl plus 81 mM MgSO_4_. Cells were grown for 72 h (OD_600_ 0.9). Bright cell inclusions are gas vesicles. The single small gas vesicle in the “empty” carbon hole (*top right*) of the grid originates from a lysed cell. The low signal-to-noise ratio of the image is due to the limited electron dose (≈60 e^−^/Å^2^), the thickness of the ice layer (>500 nm) and the contrast contribution of the salt

In a first series of experiments, we vitrified *H. salinarum* for cryo-EM by means of a commonly used manual plunger without atmospheric control (Plitzko and Baumeister 2007) taking additional care of the temperature of the cell suspension prior to plunging (for parameters and observations see Online Resource 2). Keeping cells at cultivation temperature (37 °C) instead of room temperature (RT) had no detectable improving effect on the sample quality after vitrification and was thus suspended in further tests. Blotting of grids from the sample side (grid side where the cell suspension was applied) led to cell loss while blotting from the back side turned out to be advantageous. This prevented direct contact of cells with the blotting paper while draining excess liquid through the holey carbon foil. The blotting time was critical; about 3 s gave best results whereas ≈1 s led to a very thick ice layer and ≈10 s to damage of the carbon film (for detailed results see Online Resource 2). However, the ice thickness was always highly variable and sample quality was difficult to reproduce; the conditions and procedure of the manual approach could not be sufficiently standardized. We, therefore, switched to a commercial plunger (Vitrobot™ Mark IV) and adapted the following device-controlled parameters in a series of experiments: humidity, blotting strength and time (for detailed results see Online Resource 3). Working at room temperature and 100% humidity in the sample chamber and the ability to blot from both sides provided some additional flexibility that allowed further optimization of the blotting protocol. While a 3 s blotting time always produced a thick or very thick ice layer (if combined with a low blotting strength), blotting for 7 s or longer resulted in thin or very thin ice. The best settings with an acceptable but still variable distribution of cells on the grid were a blotting time of 5 s at medium blotting strength (Online Resource 3). Cell lysis was minimal under these conditions and we only occasionally observed gas vesicles outside of microbes, indicating rupture of only a few cells in the course of the preparation process (Fig. 2).

### Vitrified *H. salinarum* cells in cryo-electron microscopy

**Fig. 3:**
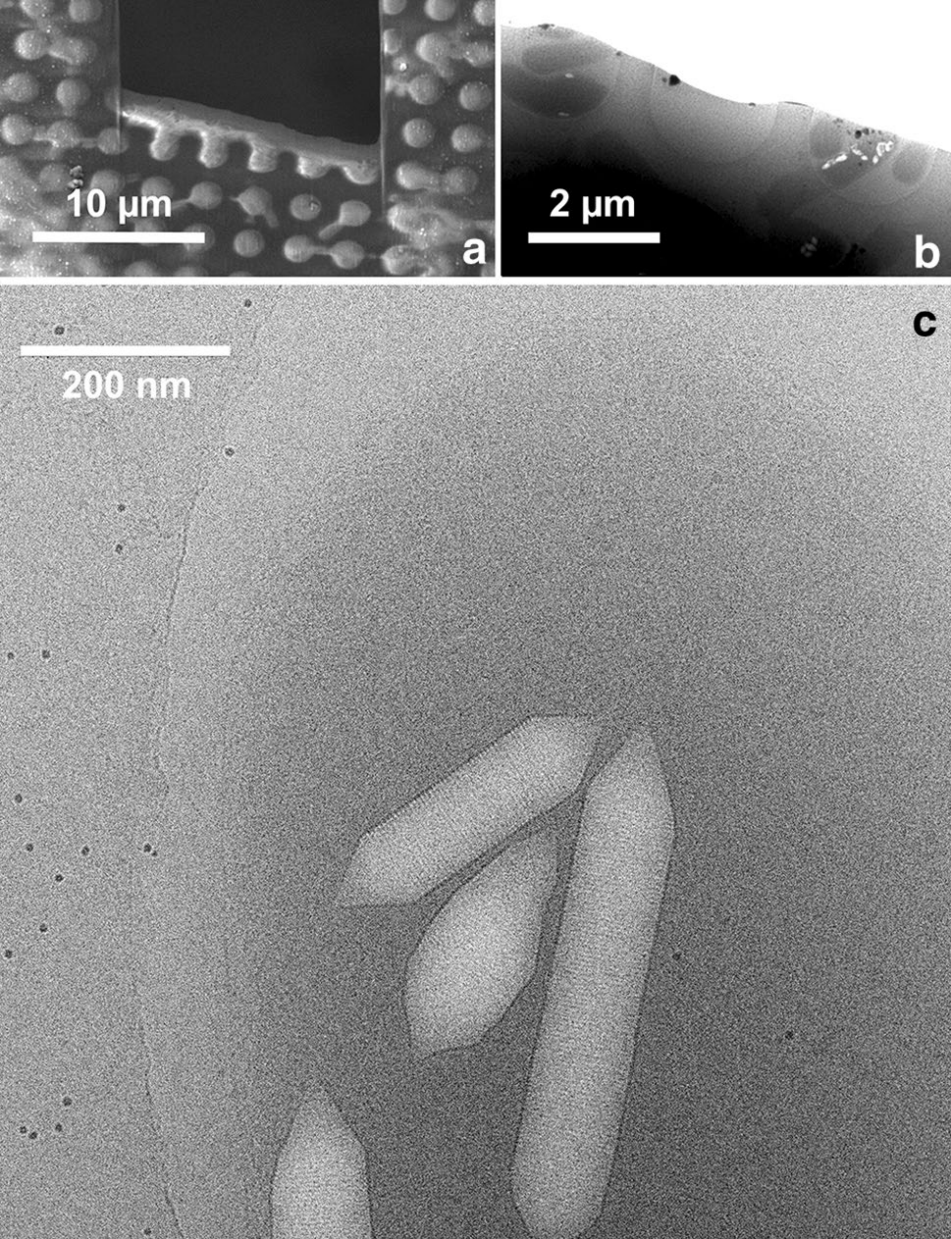
Thinning of a vitrified sample from *Halobacterium salinarum* in 3 M NaCl plus 81 mM MgSO_4_ by FIB micromachining. **a** Scanning electron micrograph of a grid region showing a wedge-like cutout. The ice layer in grid holes (*bright*) at the cutting edge is partly thinned. **b** Cryo-transmission electron micrograph of three grid holes, two of them containing thinned cells. **c** Image of a thinned *H. salinarum* cell containing gas vesicles. The periodicity of the vesicle “ribs” (4.6 nm) is clearly visible. The contrast of the cell membrane and S-layer is low due to the limited electron dose and the moderate defocus. The closest lateral distance of the individual gas vesicles to the estimated location of the cell membrane is between ≈15 and 240 nm

Most *H. salinarum* cells were between 0.5 and 0.7 µm in thickness (Fig. 2) and thus in a range that would still permit data collection for cryo-electron tomography. But the high KCl concentration inside the cytoplasm leads to considerable cellular contrast and tends to obliterate the contributions of biological structures. Cells that had lost part of their cytoplasm by limited lysis in 2.5 M NaCl occurred flattened in the microscope and showed remaining cellular details, e.g. the putative polar cap structure (Kupper et al. 1994; Metlina 2004), with reasonable contrast (Online Resource 4). We, therefore, applied cryo-FIB milling to thin intact frozen-hydrated samples in a controlled manner. Halobacteria are too small to be addressed by selective thinning as is possible and necessary for larger cells (Rigort et al. 2010; Schaffer et al. 2016). We cut wedges into the specimen covering 5–6 holes of the grid, so that cells located in these holes became concomitantly thinned (Fig. 3). The yield of individual microbes suitable for appropriate structural analysis largely depended on the distribution and orientation of cells and was difficult to predict prior to inspection in the transmission electron microscope. Importantly, while we found that cryo-FIB treatment of *H. salinarum* and cryo-EM in 3 M NaCl is technically practical, it is advisable to prepare a number of FIB milled EM grids to obtain a series of suitable data.

## Conclusions

The conjecture that cellular cryo-electron microscopy of extreme halophiles is impossible because of the exceptionally high salt requirement for cell integrity apparently excluded an interesting group of microbes from in-situ investigations. In this study, we showed that halophilic microbes can be imaged under close-to-live conditions. The crucial technical step is the adaptation of the vitrification process that turned out to be delicate and critical for obtaining healthy cells for visualization. The still remaining variability of the cell distribution and ice thickness likely is a principal problem of the badly defined blotting process (Glaeser et al. 2016). Very recently developed “self-blotting” grids may possibly help to improve the situation in the future (Razinkov et al. 2016). Nevertheless, the parameter settings evaluated here serve as a guideline for the preparation of halophiles in general. While vitrified cells have not been investigated in solutions clearly beyond 0.1 to 0.2 M salt so far, with one exception (Zenke et al. 2015), there are already a few studies on isolated viruses from halophilic microbes in 1–1.3 M NaCl (Jäälinoja et al. 2008; Aalto et al. 2012; Pietilä et al. 2013). These experiments and our own experiences suggest that cryo-EM of halophiles in media below ≈2 M salt is not exceedingly problematic; beyond that, in-situ investigations become increasingly challenging.

Salt concentrations as high as 3 M NaCl are manageable but are not ideal for routine and high throughput approaches. Gentle adaptation of cells to less salt via growth and subsequent dialysis was necessary for *H. salinarum* and might also be a promising strategy to establish convenient conditions for microscopical preparation and imaging for species with more moderate salt requirements. *Halobacterium salinarum* and other haloarchaea need divalent anions to protect the cell wall (here the S-layer) against osmotic pressure differences (Engelhardt 2007; Kessel et al. 1988). The Mg^2+^ concentration thus should be sufficiently high in corresponding experiments. But divalent anions also appear to play a role for the functional integrity of peptidoglycan in (halophilic) bacteria (Mouné et al. 2000; Kern et al. 2010).

The salinity of the external medium is not the only parameter that matters for cryo-EM. Cells accumulate salt (KCl) and/or compatible solutes such as amino acids, derivatives thereof, or other compounds to compensate osmotic effects of the saline environment (Jehlička and Oren 2013). Particularly, internal KCl in considerable amounts increases the mass density such that the contrast of biological material becomes compromised in the electron microscope. If microbes follow the high-salt-in strategy, e.g. *Halobacteriaceae, Halanaerobiales* and *Salinibacter ruber* (Oren 2008), reducing the internal concentration by growth adaptation and complementary short-term dialysis is advantageous or even indispensable for imaging intracellular structures. By this way and combined with thinning of intact cells by FIB milling, the characterization of individual halobacterial organelles in situ such as the flagellar motor together with its polar cap (Kupper et al. 1994; Metlina 2004) or the cellular morphogenesis of gas vesicles (Pfeifer 2012) should be approachable now.

## Electronic supplementary material

Below is the link to the electronic supplementary material.

Supplementary material 1 (PDF 4893 KB)
