# Supplementary material for: Cryo-electron microscopy of an extremely halophilic microbe: technical aspects
Source: Extremophiles. 2017 Jan 3;21(2):393–8. doi: 10.1007/s00792-016-0912-0 (PMC5329092; doi:10.1007/s00792-016-0912-0)
Supplement: Supplementary file 1 — Supplementary material 1 (PDF 4893 KB) [file 792_2016_912_MOESM1_ESM.pdf]

**Extremophiles**

# Cryo-electron microscopy of an extremely halophilic microbe: technical aspects

**Daniel Bollschweiler, Miroslava Schaffer, C. Martin Lawrence, Harald Engelhardt**

Max-Planck-Institut für Biochemie, Martinsried, Germany

engelhar@biochem.mpg.de

**Online Resource 1 / Supplementary Figure**

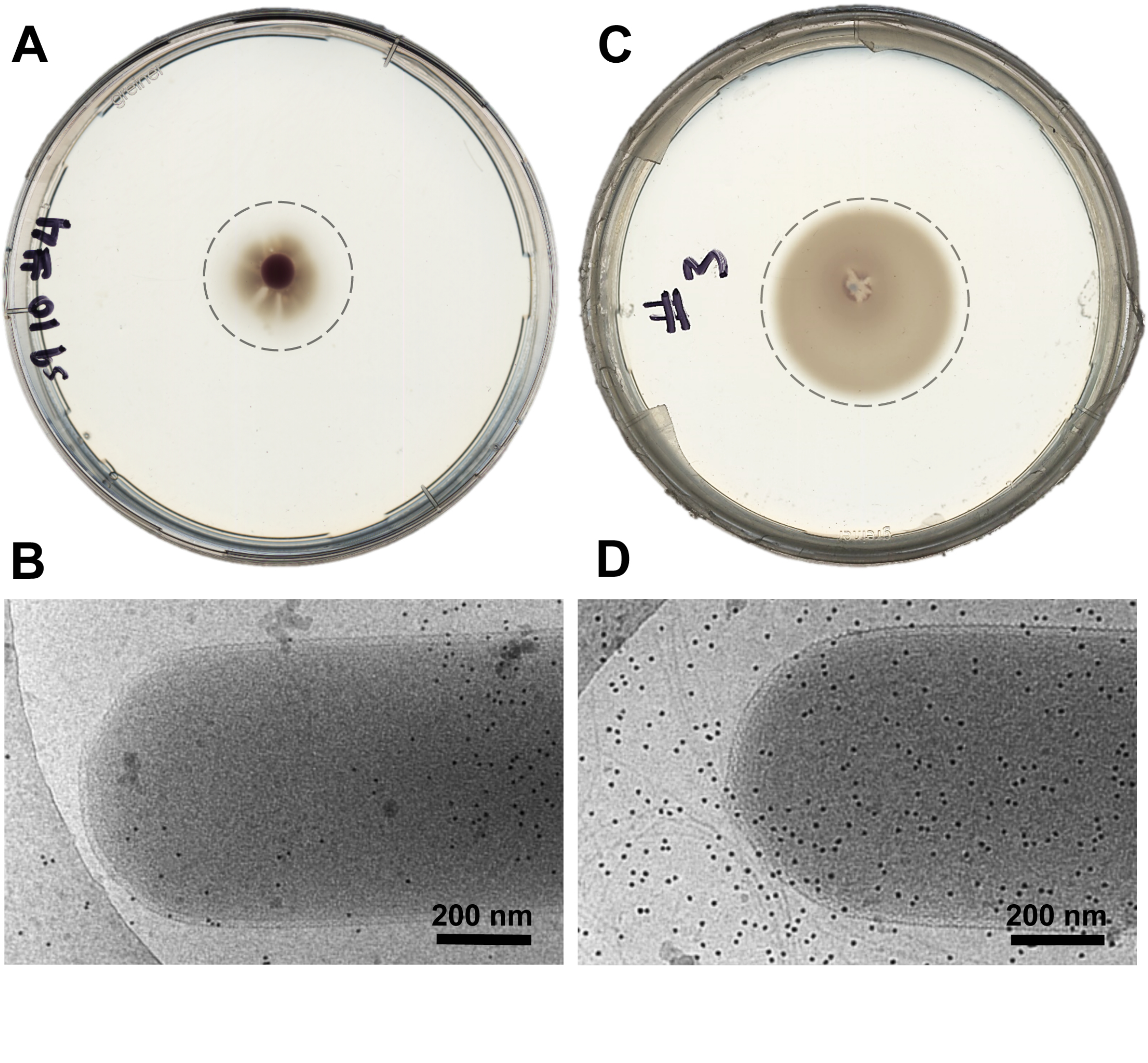


**Fig. S1** Motility and flagellation of *Halobacterium salinarum* strains. (A) Swarm agar plate incubated with strain S9 after 9 days of growth. The circle indicates the very faint border of the swarm colony. (B) Cryo-EM image of a representative cell from a sample corresponding to plate A. (C) Swarm agar plate incubated with strain SW5 selected for motile cells after 9 days of growth. (D) Cryo-EM image showing a representative cell with massive flagellation from a sample corresponding to plate C. The black dots in images B and D originate from 10 nm gold particles added to the samples.

**Online Resource 2 / Supplementary Table**

Droplets of 4 µl cell suspension of *Halobacterium salinarum* in 3 M NaCl plus 81 mM $MgSO_4$ were mixed with 4 µl colloidal gold in the same salt solution and applied to Quantifoil Cu 200 R 2/1 grids for cryo-electron microscopy. The grids were blotted and the samples vitrified by plunge freezing. Tables S1 and S2 compile the preparation parameters and the results of vitrification for manual and Vitrobot-assisted freezing experiments, respectively.

**Table S1 Manual plunge-freezing: parameters and sample quality[1)]**

| | | | | | |
|---|---|---|---|---|---|
| Cells[2)] at RT[3)] prior to dialysis | 1 min to 1 h | <10 min / 1 h | <1 min | 1 h | 1 h |
| Temperature of dialysis bath | RT / 37 °C | RT | 37 °C | RT | RT |
| Blotting from | sample side | back side | back side | back side | back side |
| Blotting duration | ≈ 3 s / ≈ 5 s | ≈ 3 s | ≈ 3 s / ≈ 5 s | ≈ 1 s | >10 s |
| Ice quality | varying thickness | varying thickness | varying thickness | very thick ice | carbon film damaged |
| Sample quality | very few cells on grid | medium to thick cell layer | few cells on grid | cells almost invisible | no cells |

[1)] Result of 4 to 8 independent tests for each condition
[2)] Cell growth 72 hours troughout
[3)] Room temperature

**Online Resource 3 / Supplementary Table**

**Table S2 Vitrobot-assisted plunge-freezing: parameters and sample quality[1)]**

| | | | | | | | | |
|---|---|---|---|---|---|---|---|---|
| Chamber humidity[2)] | 0 % | 0 % | 100 % | 100 % | 100 % | 100 % | 100 % | 100 % |
| Blotting strength[3)] | 3 / 5 | 10 / 15 | 3 | 5 | 10 / 15 | 5 | 5 | 5 |
| Blotting duration | 3 s | 3 s | 3 s | 3 s | 3 s | 5 s | 7 s | 9 s / 11 s |
| Ice quality | very thick ice | thick ice | very thick ice | thick ice | thick ice | medium ice thickness | thin ice | thin to very thin ice |
| Sample quality | good cell distribution on grid | cells often on carbon and damaged | cells invisible | good cell distribution on grid | cells mostly on carbon and damaged | very good cell distribution on grid[4)] | good cell distribution on grid | few cells on grid, cells compressed |

1) Result of 4 to 8 independent tests for each condition
2) Chamber at room temperature throughout
3) Numeric scale assigned to increasing angular range limit of the blotting arms
4) Best experimental parameter setting

**Online Resource 4 / Supplementary Figure**

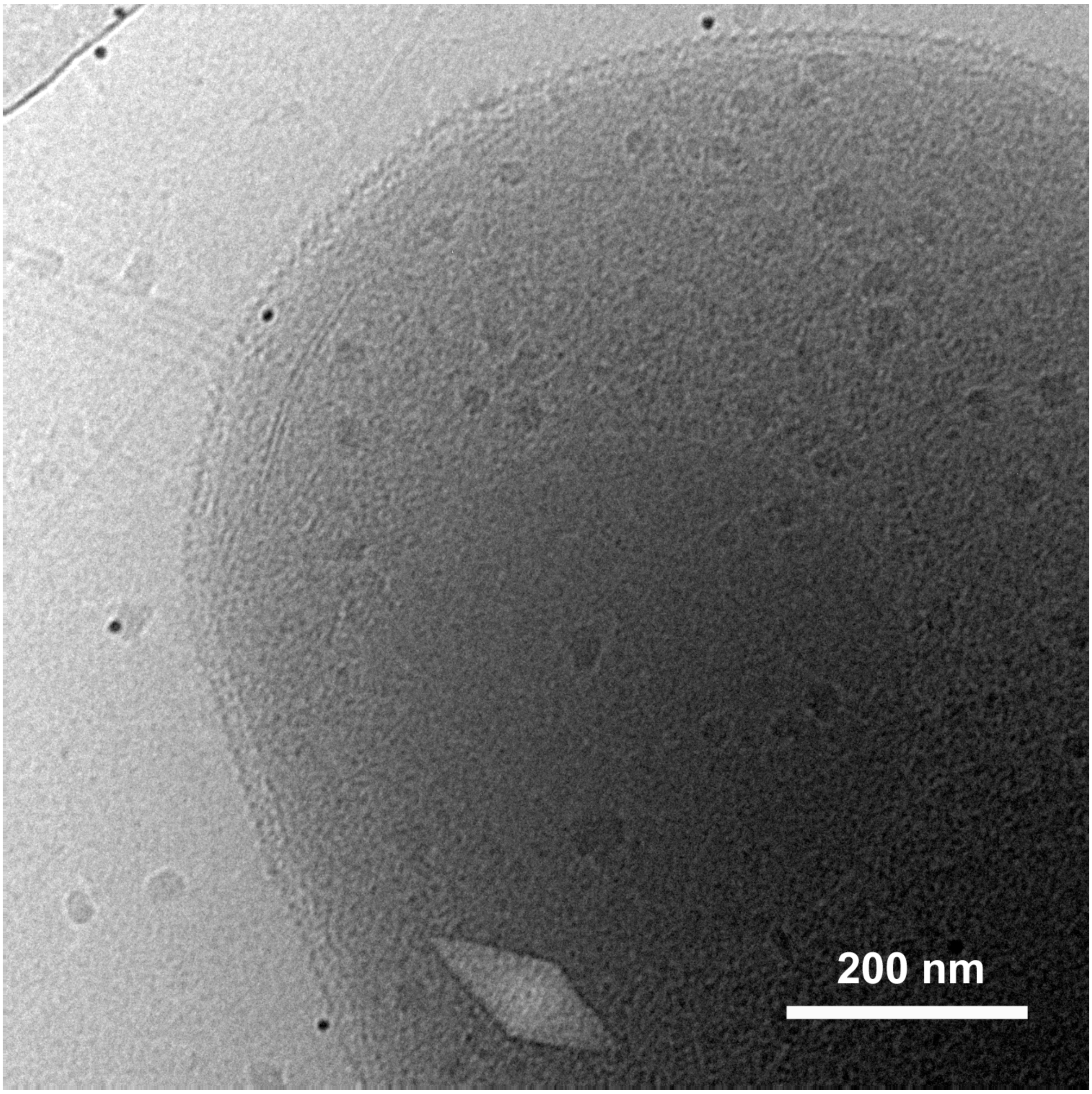


**Fig. S2** Cryo-electron micrograph of a partly lysed (flattened) cell of *Halobacterium salinarum* vitrified in a solution of 2.5 M NaCl plus 81 mM $MgSO_4$. The S-layer, cellular membrane, a bundle of flagella and the putative polar cap structure close to the cellular origin of flagella (top left of the cell) together with a small gas vesicle below a globular granule are visible. The black dots orginate from 10 nm gold particles added to the sample, the irregular gray "flakes" are ice contaminations. Projection recorded at 9 µm underfocus.
